# A comparative study on long-term evoked auditory and visual potential responses between Schizophrenic patients and normal subjects

**DOI:** 10.1186/1471-244X-11-74

**Published:** 2011-05-04

**Authors:** Min-Wei Huang, Frank Huang-Chih Chou, Pei-Yu Lo, Kuo-Sheng Cheng

**Affiliations:** 1 Institute of Biomedical Engineering, National Cheng Kung University, Tainan 701, Taiwan; 2 Kai-Suan Psychiatric Hospital, Kaohsiung 802, Taiwan; 3 Department of Psychiatry, Chiayi Branch, Taichung Veterans General Hospital, Chia-Yi 600, Taiwan

## Abstract

**Background:**

The electrical signals measuring method is recommended to examine the relationship between neuronal activities and measure with the event related potentials (ERPs) during an auditory and a visual oddball paradigm between schizophrenic patients and normal subjects. The aim of this study is to discriminate the activation changes of different stimulations evoked by auditory and visual ERPs between schizophrenic patients and normal subjects.

**Methods:**

Forty-three schizophrenic patients were selected as experimental group patients, and 40 healthy subjects with no medical history of any kind of psychiatric diseases, neurological diseases, or drug abuse, were recruited as a control group. Auditory and visual ERPs were studied with an oddball paradigm. All the data were analyzed by SPSS statistical software version 10.0.

**Results:**

In the comparative study of auditory and visual ERPs between the schizophrenic and healthy patients, P300 amplitude at Fz, Cz, and Pz and N100, N200, and P200 latencies at Fz, Cz, and Pz were shown significantly different. The cognitive processing reflected by the auditory and the visual P300 latency to rare target stimuli was probably an indicator of the cognitive function in schizophrenic patients.

**Conclusions:**

This study shows the methodology of application of auditory and visual oddball paradigm identifies task-relevant sources of activity and allows separation of regions that have different response properties. Our study indicates that there may be slowness of automatic cognitive processing and controlled cognitive processing of visual ERPs compared to auditory ERPs in schizophrenic patients. The activation changes of visual evoked potentials are more regionally specific than auditory evoked potentials.

## Background

The cognitive slowing or delay whether occurs in schizophrenic patients or not, has been debated for long time [1]. The studies of event-related brain potentials (ERPs) have shown that attributes of the ERP can be used as a dependent variable in the study of human information processing [2]. Evoked potentials from different stimulations are assumed to reflect the anatomical and functional differences between the auditory and the visual pathways. The P300 event-related potentials (ERPs) are conducted as clinical applications to detect cognitive functions. Reduction of the amplitude of the P300 component of the event-related potential (ERP) is the most replicable biological marker of schizophrenia [3]. Some meta-analytical studies strongly confirm the existence of ERP deficits in schizophrenia. Significantly magnitudes of these deficits are similar to the most robust findings reported in neuro-imaging and neuro-psychology in schizophrenia [4-7]. In the research on electrophysiological measure of schizophrenia, investigations to identify biomarkers of the disorder, indices enabling differential diagnosis among psychotic disorders, prognostic indicators or endophenotypes have been done [8]. The ERPs provide a means of measuring the cognitive processing that is independent of the motor speed and disability and reflects processes that occur between the stimulus and the response; thus, the ERPs provide the information about their courses [9,10]. The P300 is a positive ERP recorded widely across the scalp approximately 300 ms after an auditory, visual, or somato-sensory "oddball" stimulus, which must be random and stand out, and also must be followed by a response from the patient, such as pressing a button. The P300 recorded from the scalp has several components that seem to be independently generated from different brain structures. These components include brain activities involved in selective attention, work update, and short-term memory in response to unexpected changes in the environment [11,12]. The P300 latency, is presumed to indicate the time required for task evaluation independent of motor processing, can be used to study the cognitive processing in the disease. There are some reports that provide evidence of cognitive slowing or delay during auditory or visual oddball tasks by showing delayed P300 in schizophrenic patients. Roth and Cannon recorded reduced amplitude and delayed latency of the P300 waveform in patients with the disorder [13,14]. There are evidences that show increased P300 latency and reduced amplitude which are stable trait markers of risk of schizophrenia [12]. Some meta-analytical studies confirm the existence of ERP deficits in schizophrenia [5,15]. Some family study shows the P300 amplitude and especially the P300 latency are promising alternative phenotypes for genetic research into schizophrenia [6]. The P300 continues to be an important indicator of cognitive processes such as attention and working memory and of its dysfunction in neurologic and mental disorders. It has been increasingly considered as a potential genetic marker of mental disorders [16]. The presence of substantial genetic influences on schizophrenia and event-related potentials suggests that a research on neurochemical mechanisms of the abnormalities in event-related potentials may illuminate the patho-physiology of schizophrenia [17]. However, there are some studies associated with schizophrenia, combined auditory ERP with visual ERP. Schizophrenic patients are significantly impaired in their ability to form and utilize transient memory traces to guide behavior. These deficits are associated with failures of the cortical activation occurring within several hundred milliseconds after a stimulus presentation [18-20].

The aim of this study is to investigate the difference of auditory and visual long-term evoked potentials between schizophrenic patients and normal subjects. We have applied the Biologic System Company's Evoked Potential System (EP) microcomputer-based system to collect and analyze human electroencephalogram (EEG) signals. The data contains of the patient's EEG responses to two different auditory and visual stimuli using the oddball paradigm. Electrical signals measured at standard locations on the scalp were processed to detect and identify the visual and auditory ERPs in schizophrenic patients.

## Methods

### 2.1. Subjects

The study included 43 schizophrenic patients and 40 control subjects. The 43 schizophrenic patients (22 men and 21 women with age ranging from 18 to 45 years with a mean of ± SD, 27.0 ± 7.9 years) had a definite clinical diagnosis of schizophrenia according to *Diagnostic and Statistical Manual of Mental Disorders, Fourth Edition *(*DSM-IV*) criteria [15]. The patients diagnosed as a case of chronic or acute dementia according to *DSM-IV *criteria were excluded from the study. The 40 control subjects (15 men and 25 women with age ranging from 18 to 45 years with a mean of ± SD, 25.6 ± 9.2 years) had no history of psychiatric disease, neurological disease, or drug abuse. There were no differences in age, sex, marital status, and religion among subjects, but there was a significant difference in education level. All the subjects gave signed informed consent after the purpose of the study and the protocol had been informed and explained to them and before any procedure was performed. The study protocol was approved by the Hospital Ethical Committee.

### 2.2. Measurement of ERPs

The Brain Atlas III Computer of the Biologic System Company recorded the ERPs using the linked-ear reference in an auditory oddball paradigm. The system's versatility allows the user to record up to 4 sets of stimulus-evoked activity (including auditory ERP, visual ERP etc), display and analyze the data in a variety of ways. The ERPs were recorded by the surface electrodes placed in the electrode position according to the 10-10 International System with reference to both linked mastoid processes. The electrode sites were identified by Fp1, Fp2, AF3, AF4, F7, F3, Fz, F4, F8, FC5, FC1, FC2, FC6, T7, C3, Cz, C4, T8, CP5, CP1, CP2, CP6, P7, P3, Pz, P4, P8, PO3, PO4, O1, and O2. However, the used statistical method with factor analysis supports electrode positions including Fz, Cz and Pz were indicators for schizophrenia. The EOG was monitored using a forehand-temple montage with a rejection level of ± 100 μV. An electrode impedance was maintained below 5 k [ohm]. The ERPs were elicited by tone pips of 50-ms duration (10-ms rise and fall times) using the stimulation rate of 1.3/s. The infrequent (16.7%) high-pitched tones (2,000 Hz, 80 dB) were presented randomly interspersed with frequent low-pitched tones (1,000 Hz, 80 dB) binaurally. The amplifier was used by the specifications, high filter, 30; low filter; 1.0; and gain, 20,000. The time of analysis was 512 ms and the sensitivity was 122.5 mV in auditory EP testing. Subjects were asked to count the presence of infrequent high-pitched tones and ignore the frequent low-pitched tones by mental process. The error index was used to display the accuracy of the count. The artifact of vertical eyeball movement was detected from electrodes placed above and below the right eye and horizontally from electrodes placed at the left outer canthus. The data was discarded if there were more than 5 artifacts and the subjects were retested after 5 minutes.

**Figure 1:**
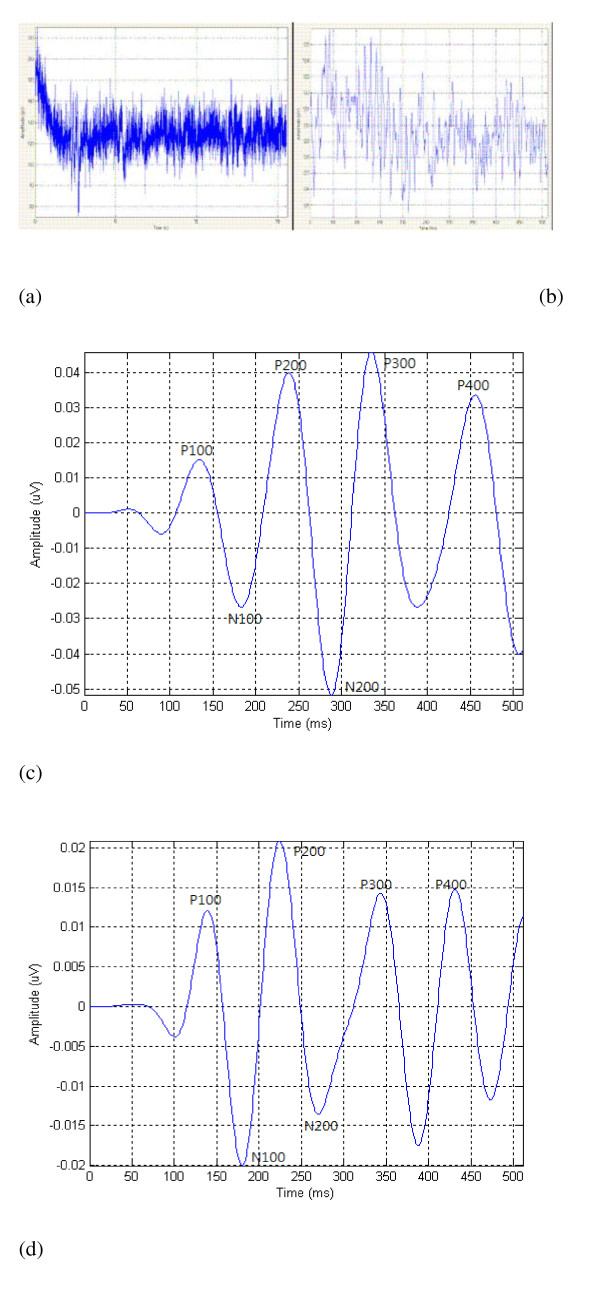
**Signal processing of evoked potential responses in control and schizophrenic groups**.(a) The EEG signals of behavioral performance at a task in which subjects had to identify and temporally order rapidly successive brief stimuli that in some trials. (b) Examples of evoked potential responses recorded in The Brain Atlas III Computer of the Biologic System Company. The system's versatility allows the user to record up to 4 sets of stimulus-evoked activity (including auditory ERP, visual ERP etc) and display and analyze the data in a variety of ways. The amplifier was used as follows: high filter, 30; low filter; 1.0; and gain, 20,000. (c)&(d) Averages were computed for the brain responses to target tones. Peak P300 amplitude, which accounts for individual variations in P300 latency, was measured as the most positive point from 250 to 400. Peak P400 amplitude, which accounts for individual variations in P400 latency, was measured as the most positive point from 400 to 500. The components of ERPs were identified as follows, P100, N100, P200, N200, P300, and P400. The figure 1c showed the averaged signals of evoked potentials from one normal control. The figure 1d showed the averaged signals of evoked potentials from one schizophrenic patient.

The subjects were seated comfortably in a dimly lit chamber with a portable eye-trek device (Olympus, FMD-20P) that was approximately 2 cm in front of their eyes. The visual oddball paradigm has a full-field, 1 × 1, square, black and white flashes, stimuli rate of1.3/s, bandpass of 30 and 1 Hz. The analysis time of 512 ms and sensitivity of 122.5 mV were used in visual EP testing. The latencies and the amplitudes of N100, N200, P100, P200, P300, and P400 waves were determined [19,20]. All the subjects were tested for four tasks; each task lasted approximately 5 minutes. The four tasks were labeled for auditory ERPs with counting, auditory ERPs without counting, visual ERPs with counting, and visual ERPs without counting respectively. An example showed that EEG signals of behavioral performance in a task in which subjects had to identify and temporally order rapid successive brief stimuli in some trials (Figure 1a &1b). The Figure 1c shows the average signals of evoked potentials from one normal control. The Figure 1d shows the average signals of evoked potentials from one schizophrenic patient.

The total averages were computed for the brain responses to target tones. The Peak P300 amplitude, which accounts for individual variations in P300 latency, was measured as the most positive point from 250 to 400. The Peak P400 amplitude, which accounts for individual variations in P400 latency, was measured as the most positive point from 400 to 500. The components of ERPs were identified and are shown in Figure 1c. The N100 was identified as a negative component (peak or notch) that occurs 70 to 150 ms after the initiation of the stimulus, with the most negative peak occurring between 70 and 150 ms at Fz, Cz, and Pz. P200 was identified as the most positive peak that occurs between N100 and 230 ms at Fz, Cz, and Pz. The N200 was also identified as the most negative peak between P200 and P300. The P300 was identified as a positive wave at Fz, Cz, and Pz, with a latency of 300 to 400 ms after the start of the stimulus. The P400 was identified as a positive wave at Fz, Cz, and Pz, with latency of 300 to 500 ms after the initiation of the stimulus. The N200 latency, P300 latency, and P400 latency were measured as the interval between each peak (or notch) and the onset of the stimulus. P300 and P400 amplitudes were defined as the voltage difference between the P300 peak and the pre-stimulus baseline [21].

### 2.3 Statistical analysis

A one-way analysis of variance (ANOVA) has been used to compare the ERPs (N100 and N200 latency and amplitude and P200, P300, and P400 latency and amplitude) between the schizophrenic patients and the healthy subjects. To avoid the type I error, all the *P *values were reported as two-tailed. *P *< 0.05 was accepted as statistically significant. All the data were analyzed by SPSS statistical software.

## Results

The average waveforms of these two groups were displayed for midline electrode sites (Fz, Cz, and Pz) in amplitude and latency (Table 1 &2). The analysis of the components of ERPs by the different stimuli (auditory and visual) with or without counting process in all subjects is shown in Tables 1 and 2. In the amplitude component of auditory ERPs, there were significant differences between N100 (Fz, Cz, Pz), N200 (Fz, Cz), P200 (Fz), and P300 (Fz, Cz, Pz) in auditory ERPs with counting group and N100 (Cz, Pz), N200 (Cz), P200 (Fz), and P300 (Fz, Cz, Pz) in auditory ERPs without counting group. In the amplitude component of visual ERPs, there were significant differences between N100 (Fz, Cz, Pz) and N200 (Fz, Cz) whereas in visual ERPs with counting group and N100 (Fz, Cz, Pz) in visual ERPs without counting group. In the control group, there were significant differences in the amplitude components of N200 (Cz) and P200 (Cz, Pz) and the latency components of N100 (Fz, Cz, Pz) and P200 (Pz) among different auditory stimuli with or without counting process. There were significant differences in the amplitude components of N200 (Fz, Cz), P200 (Fz, Cz), P300 (Cz, Pz), and P400 (Pz) among different visual stimuli with or without counting process in the control group. There were no differences seen in the latency components between visual ERPs with or without counting process in the control group. In the patient group, there were significant differences in the amplitude components of P200 (Fz, Cz, Pz) and P300 (Fz, Cz, Pz) and the latency component of N200 (Fz, Cz, Pz) among different auditory stimuli with or without counting process. There were significant differences in the amplitude component of P200 (Fz) and the latency component of P400 (Fz, Cz, Pz) among different visual stimuli with or without counting process in the patient group.

**Figure 2:**
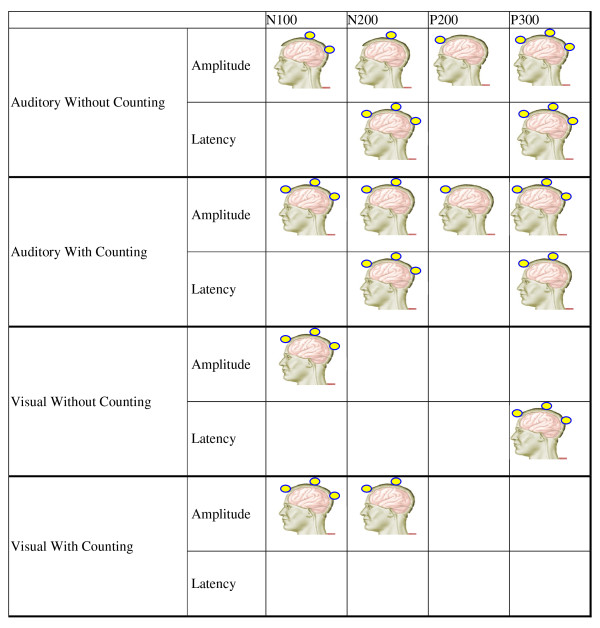
**The activation changes from latency and amplitude differences at all the scalp channels were summarized between auditory and visual event-related potentials in control and schizophrenic groups**. This difference remained significant (*p *< 0.01) for 43 schizophrenic patients and 40 control subjects after the subject-mean ERP was subtracted from each trial. The average ERP of the auditory and visual stimulus and the peaks conventionally termed N100, N200, P200 and P300. The scalp map indicates that it was obvious that the visual ERP is more specific to identify schizophrenic patients than the auditory ERP over the Fz, Cz and Pz regions.

The differences in latencies and amplitudes submitted to the ANOVA between the patient and the control groups are illustrated in Tables 1 and 2. There were no differences in latency components with either an auditory or a visual stimuli, but there was a difference seen in the P200 (Fz) amplitude component between the two stimuli (Table 1 & Table 2). The summary of latency and amplitude differences between an auditory and the visual event-related potentials in the control and the schizophrenic groups is listed in Figure 2. Figure 2 summarizes the activation changes from latency and amplitude differences at all the scalp channels between auditory and visual event-related potentials in the control and the schizophrenic groups. This difference remained significant (*p *< 0.01) for 43 schizophrenic patients and 40 control subjects after the subject-mean ERP was subtracted from each trial. The average ERP of an auditory and the visual stimulus and the peaks conventionally termed N100, P200, N200 and P300. There are more differences in amplitude and latency of ERP over N100, P200, N200 and P300 between control and schizophrenic groups after auditory oddball paradigm. Only the latency of ERP over P300, amplitude of ERP over N100, latency and amplitude of ERP over N100 and amplitude of ERP over N200 are different between the control and the schizophrenic groups after visual oddball paradigm. The scalp map indicates that the visual ERP is more specific to identify the schizophrenic patients than the auditory ERP over the Fz, Cz and Pz regions.

**Table 1:** The Amplitude Differences of Auditory and Visual Evoked-Related Potentials With and Without Counting Groups Between Control and Schizophrenic Patients

	*Auditory With Counting*			*Auditory Without Counting*			*Visual With Counting*			*Visual Without Counting*		
	**Control** **(n = 40)**	**Schizophrenia** **(n = 43)**	**P**	**Control** **(n = 40)**	**Schizophrenia** **(n = 43)**	**P**	**Control** **(n = 40)**	**Schizophrenia** **(n = 43)**	**P**	**Control** **(n = 40)**	**Schizophrenia** **(n = 43)**	**P**

**N100**												
Frontal	-2.93 (1.69)	-2.02 (1.72)	<.05	-2.94 (1.76)	-2.23 (2.01)	NS	-3.69 (1.52)	-2.46 (1.31)	<.05	-3.42 (1.93)	2.23 (1.44)	<.005
Central	-3.55 (1.92)	-2.24 (1.98)	<.005	-3.62 (1.79)	-2.58 (2.20)	<.05	-3.97 (1.69)	-2.60 (1.23)	<.05	-3.83 (2.80)	2.42 (1.32)	<.005
Parietal	-3.10 (1.80)	-2.10 (1.59)	<.005	-3.06 (1.53)	-2.22 (1.85)	<.05	-3.22 (1.63)	-1.95 (1.31)	<.05	-3.06 (1.84)	-1.88 (1.28)	<.005
**N200**												
Frontal	-3.78 (3.11)	-2.19 (1.69)	<.005	-3.06 (3.08)	-2.55 (1.82	NS	-0.59 (1.43)	-1.43 (1.60)	<.05	-1.54 (1.42)	-1.04 (1.72)	NS
Central	-4.30 (4.10)	-1.80 (1.72)	<.005	-3.26 (3.08)	-2.04 (1.89)	<.05	-0.82 (1.13)	-1.48 (1.36)	<.05	-1.66 (1.02)	-1.49 (1.54)	NS
Parietal	-2.14 (3.15)	-1.07 (1.55)	NS	-1.89 (2.05)	-1.46 (2.11)	NS	-1.00 (1.19)	-1.17 (1.31)	NS	-1.41 (0.96)	-1.47 (1.50)	NS
**P200**												
Frontal	1.20 (1.57)	1.84 (1.16)	<.05	1.47 (1.77)	2.42 (1.24)	<.005	2.05 (2.09	1.63 (2.01)	NS	2.87 (3.00)	2.18 (2.28)	NS
Central	2.02 (1.75)	2.65 (1.27)	NS	2.56 (1.87)	3.18 (1.44)	NS	2.94 (1.74)	2.79 (2.08)	NS	3.76 (1.77)	3.09 (2.21)	NS
Parietal	1.93 (1.26)	2.32 (1.14)	NS	2.42 (1.48)	2.73 (1.33)	NS	3.55 (1.55)	3.24 (1.90)	NS	3.67 (1.61)	3.33 (2.04)	NS
**P300**												
Frontal	6.28 (3.15)	3.29 (3.16)	<.000	5.81 (3.53)	2.17 (2.59)	<.000	1.69 (1.21)	2.18 (1.66)	NS	1.35 (1.61)	1.84 (1.71)	NS
Central	7.49 (4.25)	3.80 (3.25)	<.000	7.17 (4.50)	2.76 (2.66)	<.000	2.14 (1.06)	2.04 (1.46)	NS	1.48 (1.30)	1.70 (1.50)	NS
Parietal	6.96 (3.71)	3.44 (2.65)	<.000	6.59 (3.78)	2.36 (2.64)	<.000	1.80 (1.02)	1.63 (1.43)	NS-	1.21 (1.35)	1.40 (1.15)	NS
**P400**												
Frontal							1.41 (1.46)	1.99 (1.64)	NS	1.54 (1.50)	1.95 (2.26)	NS
Central							1.75 (1.14)	2.13 (1.20)	NS	1.43 (1.29)	1.77 (1.78)	NS
Parietal							1.66 (1.23)	1.79 (1.08)	NS	1.13 (1.17)	1.36 (1.67)	NS

Data are expressed as mean (SD). NS indicates not significant.

**Table 2:** The Latency Difference Of Auditory and Visual Event-Related Potentials With and Without Counting Groups Between Control and Schizophrenic Patients+

	*Auditory With Counting*			*Auditory Without Counting*			*Visual With Counting*			*Visual Without Counting*		
	**Control** **(n = 40)**	**Schizophrenia** **(n = 43)**	**P**	**Control** **(n = 40)**	**Schizophrenia** **(n = 43)**	**P**	**Control** **(n = 40)**	**Schizophrenia** **(n = 43)**	**P**	**Control** **(n = 40)**	**Schizophrenia** **(n = 43)**	**P**

**N100**												
Frontal	92.70 (14.67)	96.88 (14.92)	NS	98.65 (18.61)	98.28 (12.15)	NS	141.35 (19.14)	143.77 (20.03)	NS	140.15 (17.76)	139.81 (21.30)	NS
Central	92.80 (14.69)	96.98 (14.91)	NS	98.65 (18.61)	97.91 (12.23)	NS	141.35 (19.14)	143.67 (20.16)	NS	140.15 (17.76)	139.81 (21.23)	NS
Parietal	92.55 (14.89)	97.40 (15.10)	NS	98.70 (18.57)	97.58 (12.74)	NS	141.35 (19.14)	143.26 (20.83)	NS	140.15 (17.76)	139.77 (20.02)	NS
**N200**												
Frontal	235.70 (38.53)	263.81 (29.21)	<.000	234.60 (30.81)	280.28 (30.60)	<.000	285.90 (31.07)	292.09 (33.12)	NS	285.55 (37.72)	293.58 (39.99)	NS
Central	234.45 (39.24)	263.77 (29.72)	<.000	233.75 (31.36)	279.81 (30.99)	<.000	285.50 (30.59)	292.09 (33.12)	NS	285.55 (37.72)	293.58 (39.99)	NS
Parietal	235.00 (40.59)	264.65 (29.15)	<.000	232.95 (32.17)	279.77 (31.02)	<.000	285.50 (30.59)	292.70 (32.76)	NS	285.55 (37.72)	293.58 (39.99)	NS
**P200**												
Frontal	186.50 (35.02)	174.98 (22.06)	NS	178.65 (27.03)	174.23 (20.69)	NS	220.15 (22.79)	220.51 (27.86)	NS	220.55 (18.27)	217.44 (26.11)	NS
Central	185.85 (34.62)	174.60 (21.52)	NS	178.85 (26.63)	172.65 (20.12)	NS	219.30 (21.88)	219.72 (25.21)	NS	219.75 (18.79)	217.58 (26.00)	NS
Parietal	185.75 (34.51)	174.23 (21.74)	NS	178.00 (26.41)	173.72 (18.61)	NS	219.15 (21.90)	220.65 (24.80)	NS	218.95 (18.53)	217.63 (25.98)	NS
**P300**												
Frontal	329.75 (26.09)	344.05 (33.44)	<.05	320.85 (23.46)	341.72 (31.15)	<.005	338.95 (32.68)	351.40 (32.83)	NS	337.40 (33.21)	353.44 (31.17)	<.05
Central	329.60 (25.62)	343.53 (33.96)	<.05	322.20 (26.01)	339.86 (30.99)	<.005	338.95 (32.68)	351.95 (33.30)	NS	337.40 (33.21)	353.53 (31.24)	<.05
Parietal	330.50 (25.68)	343.49 (34.62)	NS	321.30 (25.70)	338.84 (32.27)	<.005	338.95 (32.68)	352.00 (33.14)	NS	337.40 (33.21)	354.14 (32.16)	<.05
**P400**												
Frontal							435.95 (33.29)	448.70 (29.65)	NS	435.60 (25.14)	437.49 (23.62)	NS
Central							435.70 (33.46)	448.40 (29.83)	NS	436.20 (24.64)	437.58 (22.66)	NS
Parietal							435.20 (34.23)	448.47 (30.28)	NS	436.28 (24.71)	436.74 (23.61)	NS

Data are expressed as mean (SD). NS indicates not significant.

## Discussion

Clinically, the delay of the P300 latency is a nonspecific change in psychiatric disorder. It can also be found in dementia, schizophrenia, depression, and other organic mental disorders [18,22-24]. The aim of this study is to assess whether the visual ERPs can be a clinically effective diagnostic tool to be used for differentiation of schizophrenic patients or not. Additionally, the ERPs induced by the mental process regardless of the modality of an auditory and a visual input in the same brain structures were also been examined. The paired Student *t *test is performed to compare signal processing models, assuming a unique and common mechanism as the locus of action of this effect. It includes visual or auditory ERPs with or without counting process. ERPs recorded in this process, serially presented tones or flashes, which could be in either the auditory or visual modality.

According to the detailed demographic data, there were no differences in sex, age, marital status, or religion, but there were significant differences in educational qualification. In the control group, there were no differences in the latency component of visual ERPs with or without counting, but the early N100 (Fz, Cz, Pz) and delayed P200 (Pz) in auditory ERPs with counting were noted. In the schizophrenic patients, there were no differences in the latency component of visual ERPs with or without counting except delayed P400 (Fz, Cz, Pz) in visual ERPs with counting. However, early N200 (Fz, Cz, Pz) in the auditory ERPs with counting was also noted observed. This finding shows that in either counting or without counting process the latency of visual ERPs This finding shows that in either counting or without counting process the latency of visual ERPs waswas unchangeable and unique in healthy subjects thus. This means that the latency of auditory ERPs was much more influenced by attention than visual ERPs. Otherwise, the decreased N200 (Fz, Cz), decreased P200 (Fz, Cz), increased P300 (Cz, Pz), and increased P400 (Pz) amplitude components of visual ERPs with counting but decreased P200 (Cz, Pz) in auditory ERPs with counting were noted in the control group. It is also observed that, in the case group, there were no differences in the amplitude components of visual ERPs with or without counting, but increased P200 (Fz, Cz, Pz) and P300 (Fz, Cz, Pz) in auditory stimuli the ERPs with counting were noted. This finding shows that the amplitude of visual ERPs was changed in the mental process with counting in healthy subjects but not in schizophrenic patients. This could be the result of a deficiency of signal processing in visual ERPs among schizophrenic patients. In the amplitude of auditory ERPs with counting, the P200 and P300 amplitudes increased in schizophrenic patients, proving that the signal processing enhanced by counting was observed in schizophrenic patients. In other words, the schizophrenic patients could lack abilities, such as attention, required for signal processing.

However, the P400 component exists in visual ERPs. The P400 component was identified as a positive wave at Fz, Cz, and Pz, with a latency of 300 to 500 ms after the start of the stimulus. There were no differences in the latency components in the control group with or without counting process, but there was an increased P400 (Pz) amplitude component in the control group with the counting process. There was also a significant prolonged latency of P400 (Fz, Cz, Pz) in the patient group with the visual counting process. The delayed latency of P400 in the visual counting process was observed in schizophrenic patients, which can be used for differential diagnosis clinically.

In the paired Student *t *test analysis of case and control groups, the latency components of P300 (Fz, Cz, Pz) in visual ERPs without counting or N200 (Fz, Cz, Pz) and P300 (Fz, Cz) in auditory ERPs with or without counting were significantly different between the case and control groups. This finding indicates that delayed latency of N200 and P300 in the auditory ERPs and P300 in the visual ERPs can be clinically correlated to schizophrenic patients. However, the amplitude components of N100 (Fz, Cz, Pz) and N200 (Fz, Cz) in visual ERPs with counting; N100 (Fz, Cz, Pz) in visual ERPs without counting; N100 (Fz, Cz, Pz), N200 (Fz, Cz, Pz), P200 (Fz), and P300 (Fz, Cz, Pz) in auditory ERPs with counting; or N100 (Cz, Pz), N200 (Cz), P200 (Fz), and P300 (Fz, Cz, Pz) in auditory ERPs without counting were significantly different between case and control groups. This finding implies that decreased amplitude of N100, N200 and P300 in the auditory ERPs and N100 in the visual ERPs can indicate clinical correlation among schizophrenic patients.

Various studies have shown that the amplitude of the P300 component of ERP is reduced in schizophrenic patients [25]. It is assumed that this P300 abnormality may present a disturbance in information processing required for task performance. Therefore, P300 may be an effective tool used to investigate putative neuro-biological mechanisms underlying schizophrenic symptoms [25]. Recent studies suggest that ERP measurement of auditory system adaptability characterize the pathophysiological process underlying the cognitive impairment more appropriately in schizophrenia than static measurement of ERP magnitude [26]. There are also few studies supporting the view that schizophrenia is characterized by fundamental deficits in integrative cortical functions that specifically impair the ability to analyze and represent stimulus context to guide behavior. Moreover, abnormalities of the auditory P3 amplitude in schizophrenia seem to reflect a basic underlying patho-physiological process that is present at illness onset and progresses across the illness course [27]. Our study showed decreased P300 amplitude and delayed P300 latency in auditory ERPs with or without counting but only delayed P300 latency in visual ERPs without counting between schizophrenic patients and the control group. Other studies have reported the decreased P200 latency for standard stimuli observed in the present study in both schizophrenic subjects and non-schizophrenic college students with high levels of illusory thinking. In our study, there is no significant decrease in P200 latency observed in both auditory and visual ERPs. Several previous studies have found abnormalities of N1 generation in schizophrenia [28], including both increased and decreased amplitude [29-32]. In our study, there is a significant decrease observed in N100 amplitude in schizophrenic subjects related to control and insignificant effect on N100 latency in both auditory and visual ERPs. Differences in findings regarding obligatory ERP components may relate to differences in type of stimulus (tone vs. click), intensity, duration, or interstimulus interval. We observed significantly delayed N200 (Fz, Cz, Pz) and P300 (Fz, Cz) and reduced N100 (Fz, Cz, Pz), N200 (Fz, Cz), and P300 (Fz, Cz, Pz) amplitude to auditory non-target stimuli. Visual N200 to rare target stimuli was assumed to be similar to auditory N200 and to reflect a controlled discriminative processing. In our paradigm, P300 was also thought to reflect an automatic task evaluation processing and controlled cognitive processing. The study reveals the process of cognitive delay existing in schizophrenic patients corresponding to our findings of prolonged N200 latency to auditory stimuli implies that the automatic cognitive processing could be slowed in the disease. However, there are neuropsychological studies that suggest preserved function of automatic cognitive processing in schizophrenia.

According to our study no matter what the auditory stimuli (with or without mental counting) are, the amplitude components of N100, N200, P200, and P300 and the latency components of N200 and P300 were significantly different between the control and the schizophrenic patients. However, the amplitude of N200 (Fz, Cz) induced by the visual stimuli with mental counting was significantly different between the control and the schizophrenic groups. The latency of P300 was not different between the two groups, which mean that some mental processing occurs at the N200 level during visual stimuli but that schizophrenic patients lack this ability. However, when the schizophrenic patients tried to use mental counting in the visual stimuli, the P300 latency was not different between the two groups. This indicates that the time of mental processing is not delayed among schizophrenic patients.

Because of their millisecond-level temporal resolution, ERPs are ideally suited for analysis of the brain activity related to information processing. A major finding of the present study is that the amplitude of N200 and P300 used as an index of cortical processing is delayed in schizophrenia. Mismatched negativity reflects activation of neural structures within primary auditory cortex (Heschl's gyrus) or adjacent supra-temporal auditory regions, as opposed to N200, which primarily reflects activity within auditory association cortex, and P3, which reflects activity in prefrontal, temporo-parietal, and, potentially, other multiple sensory association regions of the cortex. Our findings, therefore, indicate that the neuro-physiological dysfunction in schizophrenia is prevalent and extends even to the level of the sensory cortex [33].

An important aspect and contribution of this study is to integrate the auditory and the visual ERPs for patients with schizophrenia. The implementation of such tools may be significantly used for clinical interventions. People with schizophrenia may be followed up with such tools in the longitudinal follow-up study. Since there is no evidence of any published literature along with all meta-analyses, a caution should be taken into account while interpreting the results. Patients with schizophrenia should be considered separately for the study from those with different types if large sample size.

## Conclusions

This study demonstrated on visual ERPs indicates that there may be a slowness of automatic cognitive processing and a controlled cognitive processing in schizophrenia. The P300 latency implies that the controlled cognitive processing in schizophrenia is influenced by slower information input at mismatched negativity, which reflects activation of neural structures within primary auditory cortex (Heschl's gyrus) or adjacent supra-temporal auditory regions. The auditory and visual P300 latency can be a very powerful evaluation tool to study the condition of schizophrenia, although the auditory N100 and the visual N100 amplitude and latency may contribute to ERP results when the patients and the normal control subjects are compared. These findings can be used for future applications of N100 and P300 in the study of this particular disorder by enhancing measurement sensitivity and promoting greater clinical utility.

However, N200 primarily reflects activity within the auditory association cortex and P3 reflects activity in prefrontal, temporo-parietal and potentially other multiple sensory association regions of the cortex. This study shows how the application for auditory and visual oddball paradigm identifies task-relevant sources of activity and allows separation of regions that have characteristic response properties. The activation changes of visually evoked potentials and are more specific regionally than auditory evoked potentials are. In the clinical implications, the implementation of such tools may be significantly useful for clinical interventions. It is therefore possible to integrate the auditory and the visual ERPs for patients with schizophrenia. People with schizophrenia may be followed up by such tools in the longitudinal study in future.

## Competing interests

The authors declare that they have no competing interests.

## Authors' contributions

KSC and MWH conceived the study, designed the protocol, analyzed the data and prepared the manuscript. PYL participated in the study design and significant comments on the manuscript. FHC participated in the study design and helped to draft the manuscript. All authors have read and approved the final version of the manuscript.

## Pre-publication history

The pre-publication history for this paper can be accessed here:

http://www.biomedcentral.com/1471-244X/11/74/prepub
